# PReS-FINAL-2273: Clinical and laboratory characteristics of patients with fever of unknown origin in two Colombian pediatric rheumatology centers from 2010 to 2013

**DOI:** 10.1186/1546-0096-11-S2-P263

**Published:** 2013-12-05

**Authors:** RM Eraso, MP Velez, F Montoya, J Sierra

**Affiliations:** 1 Faculty of Medicine, Universidad de Antioquia, Medellin, Colombia

## Introduction

Fever of unknown origin (FUO) represents a diagnostic challenge and is a common cause of referral to pediatric rheumatology clinics.

## Objectives

To describe the clinical and laboratory characteristics of patients with diagnosis of FUO who were seen in two pediatric rheumatologic reference centers in Medellín, Colombia, in order to identify specific characteristics of FUO secondary to rheumatic diseases (RD).

## Methods

We included patients from a prospective diagnostic test trial called*: levels of total and glycosilated ferritin in children with systemic onset juvenile idiopathic arthritis (SoJIA) and children with other causes of FUO*. Patients had been referred with the diagnosis of FUO, defined as: temperature of >38.3°C at least twice per week during two or more weeks and without a clear diagnosis after initial evaluation. Epidemiological variables, fever characteristics and the following clinical manifestations were considered: arthritis, lymphadenopathy, evanescent rash, hepatosplenomegaly and serositis. We included the following laboratory tests: complete blood count, erythrocyte sedimentation rate (ESR), C reactive protein (CRP), Lactic Dehydrogenase (LDH), ferritin and transaminases. We considered other variables that were obtained retrospectively from medical records including: other clinical manifestations associated with fever, antibiotic treatment before admission, laboratory and diagnostic images, presence of significant organ or system dysfunction, number of patients who died and probable cause of death. Patients were followed by pediatric rheumatology for a minimum period of six months and they were grouped into categories in accordance with their final diagnosis. We used descriptive statistics. We compared the main clinical and laboratory characteristics of patients with RD versus all patients with other causes of FUO using the chi-square test for categorical variables and for continuous variables the U Mann Whitney test was applied.

## Results

53 patients were included, 60.3% were male and average age was 6 years. Median total fever duration: 30 days (ICR 21-42). 66% had received empirical antibiotics before study admittance, most of them more than one. RD were the most frequent category of FUO (51%) followed by: miscellaneous causes (15%), infections (11%), unidentified causes (11%), malignancy (6%), and hemophagocytic lymphohistiocytosis (6%). The most common of the RD was SoJIA. Arthritis, evanescent rash, serositis, neutrophilia >80% and ESR >50 mm/h were more frequent in patients with RD versus other causes of FUO (p < 0.05). An increase in LDH was more frequent in non-RD causes of FUO (p < 0.036). Of all patients, 5.7% died and 39% presented an organ or system dysfunction, the most common being hematological. No differences in fever characteristics or other clinical and laboratory variables by comparing RD with other types of FUO were found.

## Conclusion

FUO comprised a wide range of diseases. RD were the most common diagnostic category of FUO and within these the SoJIA was the main cause, with certain clinical and laboratory findings as clues to the diagnosis. Organ dysfunction was observed more frequently than in other series of FUO.

## Disclosure of interest

None declared.

